# Alcohol and khat dual use among male adults in Ethiopia: A multilevel multinomial analysis

**DOI:** 10.1371/journal.pone.0290415

**Published:** 2023-09-21

**Authors:** Kirubel Dagnaw Tegegne, Moges Muluneh Boke, Asres Zegeye Lakew, Natnael Atnafu Gebeyehu, Mesfin Wudu Kassaw

**Affiliations:** 1 Department of Nursing, College of Medicine and Health Science, Wollo University, Dessie, Ethiopia; 2 Department of Reproductive Health, College of Medicine and Health Science, University of Gondar, Gondar, Ethiopia; 3 Associate Animal Health Researcher at Sirinka Agricultural Research Center, Woldia, Ethiopia; 4 Department of Midwifery, College of Medicine and Health Science, Wolita Sodo University, Wolita Sodo, Ethiopia; 5 Department of Nursing, College of Health Science, Woldia University, Woldia, Ethiopia; Curtin University Bentley Campus: Curtin University, AUSTRALIA

## Abstract

**Introduction:**

Substance use has been a long-standing global public health problem with detrimental physical, psychological, social, and economic consequences at individual and societal levels. Large-scale and gender-specific studies on the dual use of alcohol and khat are limited. This study aimed to estimate the prevalence of dual alcohol and khat use and identify associated factors among male adults in Ethiopia.

**Methods:**

The present study used data from the 2016 Ethiopian Demographic and Health Survey (EDHS). Prior to data analysis, the data were weighted to ensure a representative sample and obtain a reliable estimate. Multilevel multinomial logistic regression was used to identify the factors associated with alcohol and khat use. Adjusted Odds Ratios (AOR) with 95% confidence interval and P value ≤ 0.05 in the multivariable model were used to identify significant factors associated with alcohol and khat use.

**Results:**

This study included 12,688 participants, of which (80.29%) were from rural areas. The mean age of participants was 30.92 years old. The prevalence of neither Alcohol nor Khat users were (33.2%); 95% Confidence Interval (CI) (32.4–34.1) only Khat users (22.0%); 95% CI (21.2–22.7), only Alcohol users (35.6%); 95% CI (34.7–36.4), and dual Alcohol and Khat users were (9.0%); 95% CI (8.5–9.5). At the individual level: being in the age group of 15–29 years and 30-49years increases the odds of Khat chewing by AOR (95%CI) 2.27 (1.75, 2.89) and 1.55 (1.16, 2.07) times, respectively. At the community level: males from Amhara 3.49(1.91, 6.42), and Tigray 2.7(1.49, 5.05) regions were more likely to drink alcohol.

**Conclusion:**

The high prevalence of dual alcohol and khat use implies for greater access to evidence-based treatment. Multiple factors are associated with alcohol and khat use at individual and community levels. All male adults would benefit from targeted preventive strategies.

## Introduction

Substance use has been a long-standing global public health problem [1]. The detrimental use of substances has been reported to have consequences on individuals, families, and societies [2]. For example, diseases; psychological, social, and economic burdens in societies, and other physical harms; such as road traffic accidents [3]. According to the 2021 World Drug Report, approximately 275 million people used substances worldwide in the preceding year, with another 36 million people diagnosed with substance use disorders globally [4]. Substance use was linked to about 18 million health spans lost in 2019 alone [5]. Moreover, 2002–2030 projections by the Global Burden of Disease project estimated that five of the six fastest-growing causes of global mortality are related to substance use [6].

Alcohol is one of the most commonly abused substances across different countries [7]. Alcohol is widely consumed in American, European, and Western Pacific regions [6, 8]. In 2018, the WHO reported that alcohol consumption contributed to more than 200 diseases and injury-related health conditions, ranging from liver diseases, road injuries, and violence, to cancers, cardiovascular diseases, suicides, tuberculosis, and HIV/AIDS [9]. In low-and middle-income countries, alcohol use disproportionately affects disability and premature death [10]. Likewise, alcohol is the most predominantly used substance in Africa.

In Ethiopia, alcohol, khat, and tobacco are the most commonly consumed substances [11, 12]. Girma et al. analyzed 12,688 male cohorts in the 2016 EDHS and found that at least 62.5% had a current history of substance use (alcohol, Kath, or tobacco) at the time of the survey. In this study, alcohol (53.1%) was reported as the most commonly used substance, followed by khat use (25.9%) [13]. Khat is a plant containing psychoactive substances. It is commonly used by chewing, but is also infused as tea, dried, and smoked like cigarrete [14]. In Ethiopia, khat is usually used for social recreation, religious rituals, and as a stimulant, particularly by students, to improve academic performance [15, 16]. A study in Eastern Africa revealed that the mean age to start khat chewing was 15.1 years [14]. Studies have shown that khat chewing is associated with heart diseases, abdominal problems, mental illness, oral health problems [17, 18], and is a cause of unstable household economies [19].

Apart from the negative health effects of khat, people who use it tend to progress toward alcohol use. A systematic review and meta-analysis in Ethiopia suggested that the pooled current and lifetime alcohol use rates were 23.86% and 44.16%, respectively [20]. Despite Ethiopian policy on khat that exhibits disapproval of its cultivation and consumption, khat continues to be one of main sources of the annual earnings. However, advertisements, and billboards promoting alcoholic drinks are forbidden. Alcoholic products shall hold a warning labels that prohibits the sell for those who are less than 18 years of age [21, 22]. Studies in Ethiopia have pointed out that the contributing factors for alcohol use were male sex [20, 23, 24], being orthodox religion followers [24, 25], smoking [26]. Furthermore, studies indicated that male sex, Muslim religion, low socioeconomic status, low educational level are the factors associated with khat consumption [27, 28].

In Ethiopia, there is still a paucity of national data regarding the epidemiology of dual alcohol and khat use across populations, with available data largely limited to separate studies investigating either alcohol or khat use. The lack of data regarding the prevalence undermines our ability to adequately understand the full extent of the problem. Despite previous studies investigated the prevalence of either alcohol or khat use in men populations [25, 29], the studies are limited to the prevalence of either alcohol or khat use in separate among male populations. To our knowledge, there are no gender-specific studies on the prevalence of dual (simultaneous) alcohol and khat use in Ethiopia. Information on the prevalence of alcohol and khat use and its associated factors provides significant input for health-related policymakers to design effective intervention strategies to minimize the use and its harmful effects. It can also be used as baseline information for future researchers. Therefore, this study aimed to assess the prevalence of dual alcohol and khat use and identify associated factors among male adults in Ethiopia.

## Methods

### Data source

The 2016 Ethiopian Demographic and Health Surveys (EDHS) data were used for further analysis. The DHS is a national survey that collects information on basic demographic and health indicators, such as family planning service use, mortality, morbidity, substance use, and maternal and child health and fertility. Men, women, children, birth, and household datasets were included in the national survey; the male data coded as MR-file in the DHS website was used. The EDHS used a two-stage stratified sampling procedure to select study participants. The datasets were used to estimate the pooled prevalence and determinants of dual alcohol consumption and Khat chewing in Ethiopia. Dependant and independent variables were generated from the men’s dataset.

## Measurement of variable

### Outcome variable

The outcome variable for this study was a multinomial category: both alcohol and khat use, only alcohol use, only Khat use and non-use of both. For analysis purpose, the outcome variable coded ‘0’ if participants were non-user of both, ‘1’ if males were using only khat, ‘2’ if males were using only alcohol, and ‘3’(dual alcohol and khat use) if the males were using both alcohol and khat.

### Explanatory variables

Based on established facts and literature review, the explanatory variables included at individual level were religion, occupation, sex of head of household (Male and Female), use of Radio, use of TV, age of head of household, age group, use of internet, Educational level (no education, primary, secondary, and higher), and number of family members, cigarette smoking (yes and no), marital status, and wealth index at the household level. Whereas residence, region and wealth index at community level was contextual variables [24, 25, 30–33]. The study participants had clustering within study districts and high variation between districts.

### Operational definition

The EDHS collects data on lifetime alcohol consumption, khat chewing, and the number of drinking days in the last 30 days. However, the data in the number of drinking days of the last 30 days has more than 50% missing values. Therefore, lifetime drinking was the variable that the outcome of this study derived. Thus, ever alcohol drinking was defined as a respondent who drinks alcohol during his lifetime. Second, ever khat chewer was defined as a respondent who chewed chat during his lifetime. Dual alcohol and khat use was developed as the third outcome if the response was yes for both the alcohol and khat questions.

Use of radio and television is labeled as “yes” if the participant had the materials in their house and otherwise labeled as “no”.

Use of internet was defined as the use of internet in the last 2 year before the data collection. The response were categorized as never; if the participants had not used internet in the last 12 month, Yes; used internet in the last 1 year, and Yes; used internet before 1 year.

Wealth status was grossly categorized into 5 major quintiles according to household assets as lowest (poorest), second (poorer), middle (middle), forth (richer), and highest (richest) [34].

Substance use: was defined as the use of one or more substances such as Khat, Alcohol, tobacco (shisha or gaya) [35].

### Statistical analysis

Before the actual statistical analysis, the data were weighted to maintain the representativeness of the survey and obtain more reliable estimates. Both Rsoftware and STATA version 16 were used to perform cross-tabulations, summary statistics, and inferential statistics. Since EDHS uses two-stage stratified cluster sampling having a hierarchical composition, a multilevel regression would be appropriate to account for cluster variation in the analysis. Hence, to consider the cluster effect in the analysis, a multilevel multinomial regression model was used to identify the association between outcome and explanatory variables, where clusters (EAs) were considered as level-2 factors.

We used generalized structural equation modeling (GSEM) using the *gsem* command in Stata version 16. Model comparison was performed using deviance between the null model (a model with no independent variable), model I (a model with only individual-level factors), model II (a model with community-level factors), and model III (a model that contains both individual and community-level independent variables). The model with the lowest deviance (model III) was the best-fit model. The colinearity of closely related variables was assessed and excluded one of the two variables when the colinearity was 0.70 and above. Second, multicollinearity has been assessed using variance inflation factor (tolerance) in the multivariate model considering a threshold of VIF 10 or 0.1 tolerance. Since all variables with a colinearity score of 0.70 and above were excluded in the colinearity test, there was no multicollinearity in the final adjusted model. Variables with a p-value of ≤ 0.25 in bi-variable logistic regression were transferred to a multivariable multinomial model. Statistical significance was set at a p-value of <0.05.

### Ethics approval and consent to participate

This further EDHS analysis used a collected data from the online library by email request in https://www.dhsprogram.com/ website. Therefore, the ethical issue were usually waived. The 2016 EDHS data were reviewed and approved by the Federal Democratic Republic of Ethiopia, Ministry of Science and Technology, and the Institutional Review Board of ICF International.

## Results

### Description of the study population

This study included 12,688 male participants and the explanatory variables had no missing values. The majority of the participants 10,187(80.29%) were from rural areas. In terms of occupation, 8,417(66.33%) participants were engaged in agricultural jobs. The mean age of male participants selected for Khat and Alcoholic drink was 30.92 years old. Above 87% (11,117) of participants had never used the internet. More than half of the participants were married 7,047 (55.54%) (Table 1).

**Table 1:** The socio-demography characteristics of 15–59 years old men in Ethiopia: The 2016 EDHS source-based study.

Variables	Frequency	Percentage	Mean± Sd.
**Residence**			
Urban	2,501	19.71	
Rural	10,187	80.29	
**Religion**			
Orthodox	5,690	44.84	
Catholic	90	0.71	
Protestant	2,748	21.66	
Muslim	3,985	31.41	
Traditional	43	0.34	
Other	132	1.04	
**Owns a house alone or jointly**			
Does not own	5,739	45.23	
Alone only	4,589	36.17	
Jointly only	2,192	17.28	
Both alone and jointly	168	1.33	
**Occupation**			
Non-working	951	7.49	
Professional/technical/managerial	631	4.98	
Clerical	97	0.76	
Sales	677	5.34	
Agriculture-employee	8,417	66.33	
Services	210	1.66	
Skilled manual	826	6.51	
Unskilled manual	279	2.20	
Others	600	4.73	
**Ever been married or in union**			
No	4,895	38.58	
Formerly married	7,471	58.88	
Formerly lived with a woman	323	2.54	
**Number of unions**			
Once	5,668	72.73	
More than once	2,126	27.27	
**Current marital status**			
Never in union	4,895	38.58	
Married	7,047	55.54	
Living with partner	423	3.34	
Widowed	45	0.35	
Divorced	232	1.83	
Separated	46	0.36	
**Wealth index**			
Poorest	2,009	15.83	
Poorer	2,318	18.27	
Middle	2,443	19.25	
Richer	2,727	21.49	
Richest	3191	25.15	
**Use of internet**			
Never	11,117	87.62	
Yes, in the last 12 months	1,452	11.44	
Yes, before the last 12 months	119	0.94	
**Literacy status**			
Can’t read and write	4,082	32.17	
Able to read only parts of sentence	2,178	17.17	
Able to read whole sentence	6,368	50.19	
No card with required language	51	0.40	
Visually impaired	9	0.07	
**Educational-level**			
No education	3,840	30.26	
Primary	5,901	46.51	
Secondary	1,846	14.55	
Higher	1,101	8.67	
**Age**			30.92(30.71,31.12) ±11.60

### Difference between categories in using khat and alcohol

Among all participants, 4,169(33.2%); 95% CI (32.4–34.1) never used both alcohol and khat, 2,758(22.0%); 95% CI (21.2–22.7) participants used khat, 4,462(35.6%); 95% CI (34.7–36.4) participants used alcohol, and 1,137(9.0%); 95% CI (8.5–9.5) individuals used both alcohol and khat. In the statistical tests and inferential statistics, 12,526 male participants were involved. For the outcome variable, there were 1.28% (162) missing observations.

All the categorical variables considered in the cross-tabulation using X^2^-test have a statistical significant difference between them, except for the use of radio; there was no difference between radio users and non-users in using khat and alcohol or either of these substances (Table 2). The use of khat and alcohol in males was different among smokers and non-smokers with X^2^-test statistics value of 11.39 and a p-value of less than 0.001. Similarly, alcohol and Khat use was differed among different age groups, young age, middle adult and late adult age with a statistical X^2^-test, and significant p-value (18.43,<0.001).

**Table 2:** Distribution of dual alcohol and khat use in Ethiopia among 15–59 years old men: The 2016 EDHS source based study.

Variables	**Joint Khat and Alcohol use**				**Total (%)**	**X** ^**2**^	**P-value**
	**Neither Khat nor Alcohol use**	**Only Khat use**	**Only Alcohol use**	**Both Khat & Alcohol**			
Age					Total (%)		
Age							
15–29	2648(19.94)	1155(8.97)	2232(19.44)	469(2.69)	6504(51.03)	18.43	<0.001
30–44	1028(8.80)	1134(8.00)	1461(13.39)	471(2.94)	4094(33.13)		
45–49	493(4.27)	469(3.38)	769(7.02)	197(1.16)	1928(15.84)		
No of wives							
No wife	179 (1.18)	157(1.12)	93(0. 95)	22 (0.23)	451(3.47)	25.10	<0.001
One wife	2287(16.78)	746(5.71)	1916(16.30)	502(2.54)	5451(41.34)		
>one wives	1703(15.05)	1855(13.51)	2453(22.6)	613(4.03)	6624(55.19)		
Use of internet							
No	736(7.22)	429(3.57)	911(7.26)	254(1.50)	2330(19.55)	2.95	<0.05
Yes	3433 (25.79)	2329(16.78)	3551 (32.59)	883 (5.29)	10,196(80.45)		
Wealth index (HH level)							
Poor	1651(10.48)	1256(9.51)	1504(12.51)	185(1.42)	4596(33.92)	11.48	<0.001
Middle	558(6.26)	394(4.38)	683(7.64)	90(1.00)	1725(19.27)		
Rich	1960(16.27)	1108(6.46)	2275(19.7)	862(4.37)	6205(46.81)		
Marital status							
Single	66 (0.36)	76(0.36)	148(1.49)	77(0.30)	367(2.51)	34.00	<0.001
Divorced	2221(16.43)	670(5.35)	1768(14.81)	425(2.23)	5084(38.83)		
Married	1882(16.23)	2012(14.64)	2546(23.54)	635(4.26)	7075(58.66)		
Owns a House							
Yes	3413 (27.08)	2468 (18.74)	3604(34.36)	737(5.06)	10222(85.24)	21.54	<0.001
No	756(5.93)	290(1.61)	858(5.49)	400(1.73)	2304(14.76)		
Residence							
Urban	1175(6.35)	670(2.23)	1293(8.24)	716(3.00)	3854(19.82)	14.70	<0.001
Rural	2994(26.66)	2088(18.12)	3169(31.61)	421(3.79)	8672(80.18)		
Religion							
Orthodox	677 (5.19)	115(0. .67)	3928(35.6)	735(3.62)	5455(45.08)	206.76	<0.001
Protest	1833 (9.41)	2578 (18.94)	111 (0. 77)	228 (1.97)	4750 (31.09)		
Muslim	1577(17.63)	46 (0.37)	327 (2.69)	150 (1.10)	2100 (21.8)		
Others	82(0.79)	19 (0.36)	96 (0.78)	24 (0.10)	221 (2.03)		
TV							
Yes	3938 (30.77)	2589 (18.88)	4203 (37.92)	1102 (6.53)	11832 (94.10)	3.40	<0.05
No	231(2.25)	169(1.47)	259(1.93)	35(0.26)	694(5.90)		
Radio							
Yes	3972(31.62)	2666(19.6)	4208(37.84)	1071(6.42)	11917(95.48)	1.28	0.28
No	197(1.39)	92(0. .75)	254(2.00)	66(0.37)	609(4.52)		
Currently working							
No	253(1.54)	60 (0.13)	233(1.44)	63(0. 28)	609(3.40)	9.34	<0.001
Yes	3916 (31.47)	2698(20.21)	4229 (38.4)	1074(6.51)	11917(96.6)		
Occupation							
No	221(1.24)	48(0. 07)	147(0. 98)	40(0. 16)	456(2.45)	14.11	<0.001
Yes	3948(31.77)	2710(20.28)	4315(38.87)	1097(6.63)	12070(97.55)		
Smoking							
No	1146(9.83)	517(3.84)	1399(10.09)	450(2.37)	3512(26.13)	11.39	<0.001
Yes	3023(23.18)	2241(16.51)	3063(29.76)	687(4.43)	9014(73.87)		

### Model diagnosis and statistical association of independent variables with outcome variable

**Fig 1:**
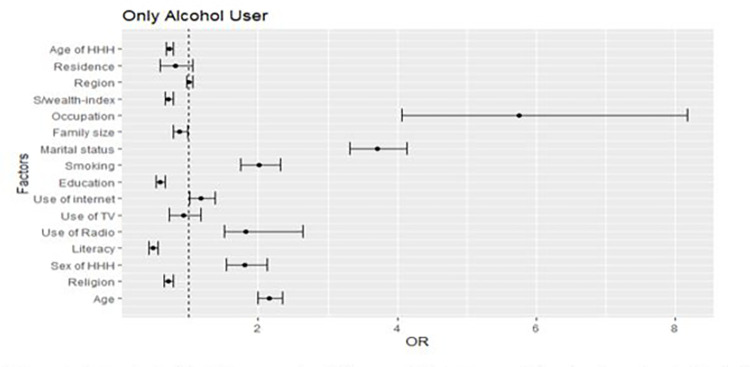
A forest plot that shows a univariable association between determinants and only alcohol users.

**Fig 2:**
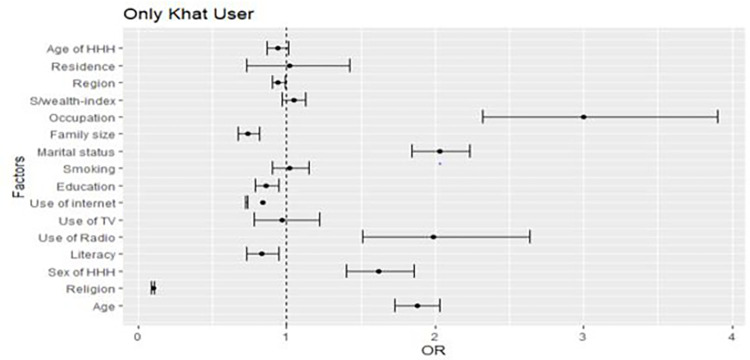
A forest plot that shows a univariable association between determinants and only khat users.

**Fig 3:**
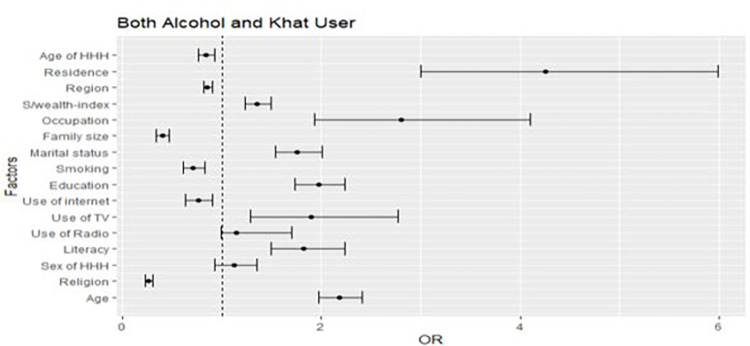
A forest plot that shows a univariable association between determinants and dual alcohol and khat users.

In the full generalized structural equation multinomial mixed effect model, age, household head age, educational level, marital status, internet use, smoking, number of family members, religion, occupation, and use of TV associated with alcohol and khat use at the individual level. At the community level, all three considered contextual variables, namely, residence, region, and community-level wealth index associated with alcohol and khat use. The variables for multilevel multinomial logistic regression were selected by a p-value of 0.25 and less in the univariable association model (Figs 1–3).

### Association of variables with only khat users, in reference to neither alcohol nor khat users

At the individual level (level-one): being in the age group of 15–29 years and 30–49 years increased the odds of khat chewing by AOR (95% CI) 2.27(1.75, 2.89) and 1.55(1.16, 2.07) times respectively, compared to 45–59 years old males. The odds of khat chewing were also higher in cigarette smokers by 51% (OR, 95%CI, 1.51(1.15, 1.97) compared to non-smokers. Having an occupation (2.43 (1.57, 3.78)) compared to people without, and being a Protestant religion (8.41(1.86, 11.13)) follower compared to orthodox religion follower, increased the odds of khat chewing. However, the age of the head of household; 15–29 years old 0.65(0.51, 0.83) decreased the odds of khat chewing by 35% compared to 45 years and above the head of household.

Similarly, divorced males 0.36(0.22, 0.56) compared to single, internet use 0.63(0.47, 0.84) compared to non-user, having a family size of 6 and above 0.82(0.70, 1.04) compared to 5 and lower, Muslim religion 0.11 (0.08, 0.17) compared to orthodox, and use of TV 0.68(0.47, 0.98) compared to non-user decreased the odds of khat chewing (Table 3).

**Table 3:** Multilevel multinomial multivariable logistic regression analysis of alcohol use, khat use, and dual alcohol and khat use among adult men by individual level correlates from 2016 EDHS data.

Variables		Model I (Null)	Model II (AOR(95%CI))	Mode III (AOR(95%CI)	Model IV (AOR(95%CI)
Reference category		Neither Chat nor Alcohol user			
Individual level variables					
Age					
Only Khat user	15–29			Reference	Reference
	30–44			2.39(1.88,3.00)	2.27(1.75,2.89)***
	45–59			1.70(1.28,2.27)***	1.55(1.16,2.07)**
Only Alcohol user	15–29			Reference	Reference
	30–44			1.48(1.16,1.86)**	1.75(1.38,2.22)***
	45–59			1.25(0.93,1.65)	1.62(1.20,2.18)**
Both Chat and Alcohol user	15–29			Reference	Reference
	30–44			4.01(3.07,5.21)***	3.07(2.34,3.97)***
	45–59			3.35(2.41,4.66)***	2.32(1.66,3.25)***
Age of head of household					
Only Khat user	15–29			Reference	Reference
	30–44			0.64(0.50,0.82)***	0.65(0.51,0.83)**
	≥45			0.78(0.61,1.003)*	0.87(0.68,1.12)
Only Alcohol user	15–29			Reference	Reference
	30–44			0.84(0.66,1.07)	0.81(0.63,1.43)
	≥45			0.98(0.78,1.23)	0.88(0.69,1.13)
Both Chat and Alcohol user	15–29			Reference	Reference
	30–44			0.56(043,0.74)***	0.62(2.14,0.82)**
	≥45			0.75(0.58,1.01)*	0.83(0.62,0.92)
Educational-level					
Only Khat user	No education			Reference	Reference
	Basic education			1.23(0.89,1.42)	0.99(0.79,1.26)
	Higher education			1.80(1.30,2.48)**	1.35(0.96,1.90)
Only Alcohol user	No education			Reference	Reference
	Basic education			0.66(0.52,0.84)**	0.86(0.67,1.12)
	Higher			0.90(0.66,1.23)	1.4(1.01,1.97)*
Both Khat and Alcohol user	No education			Reference	Reference
	Basic education			2.34(1.72,3.22)***	1.68(1.22,2.32)**
	Higher education			5.31(3.63,7.77)***	3.10(2.10,4.57)***
Marital status					
Only Khat user	Single			Reference	Reference
	Divorce			0.36(0.23,0.58)***	0.36(0.22,0.56)***
	Married			1.14(0.75,1.75)	1.22(0.79,1.88)
Only Alcohol user	Single			Reference	Reference
	Divorce			0.43(0.28,0.68)**	0.5(0.32,0.80)**
	Married			0.97(0.63,1.48)	0.74(0.62,1.51)
Both Khat and Alcohol user	Single			Reference	Reference
	Divorce			0.28(0.18,0.44)***	0.25(0.16,0.39)***
	Married			0.46(0.30,0.71)**	0.51(0.33,0.79)**
Use of internet					
Only Khat user	No			Reference	Reference
	Yes			0.67(0.50,0.89)**	0.63(0.47,0.84)**
Only Alcohol user	No			Reference	Reference
	Yes			0.97(0.76,1.23)	1.22(0.95,1.58)
Both Khat and Alcohol user	No			Reference	Reference
	Yes			0.93(0.70,1.22)	0.91(0.70,1.20)
Smoking					
Only Khat user	No smoking			Reference	Reference
	Smoking			1.48(1.14,1.93)***	1.51(1.15,1.97)**
Only Alcohol user	No smoking			Reference	Reference
	Smoking			1.03(0.83,1.30)	0.81(0.64,1.02)
Both Khat and Alcohol user	No smoking			Reference	Reference
	Smoking			0.98(0.76,1.22)***	1.16(0.90,1.49)
No of family members					
Only Khat user	≤5			Reference	Reference
	≥6			0.82(0.71,0.95)	**0.82**(0.70,1.04)*
Only Alcohol user	≤5			Reference	Reference
	≥6			1.00(0.86,1.16)	0.90(0.77,1.05)
Both Khat and Alcohol user	≤5			Reference	Reference
	≥6			0.61(0.51,0.73)	0.74(0.61,0.89)**
Religion					
Only Khat user	Orthodox			Reference	Reference
	Protest			8.41(6.49,10.91)***	8.41(1.86,11.13)***
	Muslim			0.12(0.08,0.18)***	0.11(0.08,0.17)***
	Others			1.80(0.98,3.32)	1.40(0.74,2.64)
Only Alcohol user	Orthodox			Reference	Reference
	Protest			0.01(0.007,0.01)**	0.01(0.01,0.02)***
	Muslim			0.03(0.02,0.04)**	0.04(0.03,0.05)***
	Others			0.26(0.17,0.39)***	0.35(0.23,0.53)***
Both Khat and Alcohol user	Orthodox			Reference	Reference
	Protest			0.16(0.13,0.20)***	0.21(0.17,0.27)***
	Muslim			0.07(0.05,0.09)***	0.07(0.05,0.09)***
	Others			0.46(0.26,0.80)*	0.35(0.20,0.62)***
Literacy					
Only Khat user	Can’t read			Reference	Reference
	Read			0.10(0.80,1.23)	0.98(0.79,1.22)
Only Alcohol user	Can’t read			Reference	Reference
	Read			1.03(0.76,1.21)	0.99(0.79,1.26)
Both Khat and Alcohol user	Can’t read			Reference	Reference
	Read			1.05(0.79,1.39)	0.99(0.73,1.2)
Occupation					
Only Khat user	Had occupation			1.99(1.30,3.03)**	2.43(1.57,3.78)***
	Had no occupation			Reference	Reference
Only Alcohol user	Had occupation			2.10(1.51,2.94)***	1.88(1.31,7.24)**
	Had no occupation			Reference	Reference
Both Khat and Alcohol user	Had occupation			1.91(1.26,2.91)**	2.05(1.34,3.13)**
	Had no occupation			Reference	Reference
Sex of head of household					
Only Khat user	Female			Reference	Reference
	Male			1.12(0.92,1.35)	1.17(0.96,1.42)
Only Alcohol user	Female			Reference	Reference
	Male			1.15(0.95,1.39)	0.98(0.80,1.18)
Both Khat and Alcohol user	Female			Reference	Reference
	Male			0.83(0.66,1.03)	0.89(0.71,1.10)
Radio					
Only Khat user	No			Reference	Reference
	Yes			1.48(0.99,2.16)	1.45(0.98,2.14)
Only Alcohol user	No			Reference	Reference
	Yes			1.08(0.82,1.49)	1.20(0.86,1.66)
Both Khat and Alcohol user	No			Reference	Reference
	Yes			1.06(0.73,1.54)	0.97(0.67,1.40)
TV					
Only Khat user	No			Reference	Reference
	Yes			0.71(0.50,0.98)	0.68(0.47,0.98)*
Only Alcohol user	No			Reference	Reference
	Yes			0.76(0.55,1.06)	0.91(0.64,1.28)
Both Khat and Alcohol user	No			Reference	Reference
	Yes			1.79(1.15,2.77)	1.35(0.86,2.11)

At the community level (level-two); living in Dire-Dawa 2.80(1.54, 5.10) and Harari 5.75(3.03, 10.80) regions increased the odds of khat chewing compared to men living in Addis Ababa. However, living in Afar 0.21(0.11, 0.44), Benishangul 0.25(0.13, 0.48), Somalia 0.30(0.16, 0.55), and Tigray 0.41(0.17, 0.96) regions decreased the odds of khat chewing compared to Addis-Ababa men residents. Similarly, rich communities (wealth index at community level) 0.79(0.65, 0.97) decreased Khat chewing probability by 21% compared to poor communities (Table 4).

**Table 4:** Multilevel multinomial multivariable logistic regression analysis of alcohol use, khat use, and dual alcohol and khat use among adult men by community level correlates and model diagnosis from 2016 EDHS data.

Variables		Model I (Null)	Model II (AOR(95%CI))	Mode III (AOR(95%CI)	Model IV (AOR(95%CI)
Reference category					Neither Chat nor Alcohol user
Contextual level variables					
Residence					
Only Khat user	Rural		Reference		Reference
	Urban		0.31(0.22,0.43)***		0.73(0.53,1.02)
Only Alcohol user	Rural		Reference		Reference
	Urban		1.08(0.78,1.49)		0.55(0.39,0.77)**
Both Chat and Alcohol user	Rural		Reference		Reference
	Urban		2.59(1.82,3.63)***		1.38(0.96,1.95)
Wealth index by residence (community level)					
Only Khat user	Poor		Reference		Reference
	Middle		0.87(0.73,1.04)		1.02(0.83,1.26)
	Rich		0.54(0.46,0.64)***		0.79(0.65,0.97)*
Only Alcohol user	Poor		Reference		Reference
	Middle		1.04(0.88,1.23)		0.96(0.78,1.18)
	Rich		1.05(0.90,1.23)		0.77(0.64,0.93)**
Both Chat and Alcohol user	Poor		Reference		Reference
	Middle		1.25(1.00,1.57)		1.07(0.84,1.36)
	Rich		1.28(1.05,1.58)*		0.94(0.76,1.18)
Region					
Only Khat user	Addis-Ababa		Reference		Reference
	Afar		0.28(0.14,0.53)***		0.21(0.11,0.44)***
	Amhara		1.05(0.54,2.03)		0.84(0.43,1.65)
	Benishangul		0.28(0.14,0.55)***		0.25(0.13,0.48)***
	Dire-Daw		2.75(1.49,5.05)**		2.80(1.54,5.10)**
	Gambella		0.10(0.05,0.20)***		0.64(0.32,1.28)
	Harari		5.00(2.64,9.39)***		5.75(3.03,10.80)***
	Oromia		0.72(0.39,1.32)		1.06(0.58,1.93)
	SNNPR		0.10(0.05,0.18)***		0.55(0.29,0.94)
	Somalia		0.45(0.24,0.82)**		0.30(0.16,0.55)***
	Tigray		0.20(0.09,0.45)***		0.41(0.17,0.96)*
Only Alcohol user	Addis-Ababa		Reference		Reference
	Afar		0.02(0.01,0.04)***		0.05(0.02,0.11)***
	Amhara		6.70(3.67,12.43)***		3.49(1.91,6.42)***
	Benishangul		0.56(0.30,1.04)		0.97(0.52,1.79)
	Dire-Daw		0.18(0.10,0.033)***		0.22(0.12,0.41)***
	Gambella		0.29(0.16,0.53)***		0.43(0.24,0.78)**
	Harari		0.15(0.07,3.46)***		0.14(0.07,0.28)***
	Oromia		0.26(0.14,0.46)***		0.40(0.23,0.70)**
	SNNPR		0.11(0.06,0.19)***		0.20(0.11,0.36)***
	Somalia		0.003(0.001,0.001)***		0.02(0.01,0.05)***
	Tigray		8.85(4.76,16.44)***		2.7(1.49,5.05)**
Both Chat and Alcohol user	Addis-Ababa		Reference		Reference
	Afar		0.05(0.02,0.10)***		0.06(0.03,0.12)***
	Amhara		1.99(1.04,3.82)*		1.08(0.57,2.03)
	Benishangul		0.41(0.21,1.26)**		0.41(0.22,0.79)**
	Dire-Daw		0.61(0.34,1.10)		0.61(0.34,1.09)
	Gambella		0.53(0.27,0.95)*		0.80(0.44,1.48)
	Harari		0.70(0.36,1.03)		0.53(0.28,1.01)
	Oromia		0.28(0.15,0.52)***		0.37(0.20,0.66)**
	SNNPR		0.17(0.09,0.32)***		0.33(0.18,0.60)***
	Somalia		0.02(0.007,0.04)***		0.03(0.01,0.07)***
	Tigray		1.13(0.56,2.29)		0.44(0.22,0.88)*
Random effect(clustering by the study district)					
	Variance	3.43	1.67	2.27	1.37
	ICC (%)	51.04	33.67	40.83	29.40
	PCV (%)	Reference	51.31	33.82	60.06
	MOR	2.77	3.42	4.21	3.04
IOR	Neither Chat nor Alcohol user	Reference	Reference	Reference	Reference
	Only Khat user	(0.03, 25.88)	(0.23,24.69)	(0.01, 1.92)	(0.02, 1.32)
	Only Alcohol user	(0.05, 41.87)	(0.24, 25.45)	(0.47, 110.69)	(1.26, 87.29)
	Both Chat and Alcohol user	(0.01, 10.67)	(0.04, 4.77)	(0.03, 6.94)	(0.15, 10.66)
	Model diagnostic				
	Deviance	28,774.62	23,016.98	18915.11	17290.18
	LR test	Reference	(9859.51)***	(4101.88),***	(5726.80)***
	AIC	28782.62	23102.98	19037.11	17490.18
	BIC	28812.36	23422.71	19490.68	18233.74

**Note:** AIC- Akaike’s information criterion, AOR- Adjusted odds ratio, BIC-Schwarz’s Bayesian information criteria, CI- Confidence interval, ICC- Intra-class correlation, LR test- likelihood ration test, MOR- Median odds ratio, PCV- Proportional change in variance

* P<0.05

** P<0.01

*** P<0.001.

### Association of variables with only alcohol users, in reference to neither alcohol nor khat users

In level one; the age group of 15–29 years AOR (95%CI) 1.75(1.38, 2.22) and 30–49 years 1.62(1.20, 2.18) are more likely to drink alcohol compared to 45–59 years. Similarly, higher educational level 1.4 (1.01, 1.97) compared to no education, and having occupation 1.88(1.31, 7.24) compared to people without occupation, increased the odds of drinking alcohol. However, divorced males 0.5(0.32, 0.80) compared to single males; Protestant 0.01(0.01, 0.02), Muslim 0.04(0.03, 0.05), and other religion follower males 0.35(0.23, 0.53) compared to orthodox religion followers have lower likelihood of alcohol drinking (Table 3).

At level two; males from Amhara 3.49(1.91, 6.42), and Tigray 2.7(1.49, 5.05) regions were more likely to drink alcohol compared to Addis-Ababa male residents. However, rich communities (wealth index at community level) 0.77(0.64, 0.93) decreased the odds of alcohol consumption by 33% compared to poor communities.

Similarly at community level, rural residence 0.55(0.39, 0.77) compared to urban, Afar 0.05(0.02, 0.11) Dire-Diwa 0.22(0.12, 0.41) Gambella 0.43(0.24, 0.78) Oromia 0.40(0.23, 0.70) SNNPR 0.20(0.11, 0.36) and Somalia 0.02(0.01, 0.05) regions resident were less likely to drink alcohol compared to Addis Ababa dwellers (Table 4).

### Association of variables with both khat and alcohol users, in reference to neither khat and alcohol users

At the individual level; being in the age group of 15–29 years old 3.07(2.34, 3.97) and 30–49 years old 2.32(1.66, 3.25) increased the odds of both khat and alcohol use compared to 45–59 years old. Similarly, males with a higher level of education 3.10(2.10, 4.57), basic education 1.68(1.22, 2.32) compared with no education and males with occupation 2.05(1.34, 3.13) compared to those without occupation are more likely to use both alcohol and khat. However, age of head of household, 15-29years old 0.62(2.14, 0.82) decreased the odds of both alcohol and khat use by 38% compared to heads of households aged 45 years and above. At the same level (level one), divorced males 0.25(0.16, 0.39), married males 0.51(0.33, 0.79) compared with single males, having 6 and more families 0.74(0.61, 0.89) compared with 5 and a low number of families were protective factors against both alcohol and khat use. Being a Protestant 0.21(0.17, 0.27), Muslim 0.07(0.05, 0.09), and other religions 0.35(0.20, 0.62) follower had lower odds of using both Khat and alcohol compared with orthodox religion followers (Table 3).

At level two; contextual factors mainly males living in certain regions such as; Afar 0.06(0.03, 0.12), Benishangul 0.41(0.22, 0.79), Oromia 0.37(0.20, 0.66), SNNPR 0.33(0.18, 0.60), Somalia 0.03(0.01, 0.07), and Tigray 0.44(0.22, 0.88) are less likely to use both khat and alcohol compared with Addis-Ababa (Table 4).

## Discussion

In this study, we estimated the levels of alcohol and khat use and identified associated factors among male adults in Ethiopia. The outcome variable was categorized into nominal categories (never using both alcohol and khat, only alcohol use, only khat use, and both alcohol and khat use).

We found that 22.0% 95% CI (21.2–22.7) of participants used khat, 35.6% 95% CI (34.7–36.4) used alcohol, and 9.0% 95% CI (8.5–9.5) used both alcohol and khat. The estimated khat use in our study is higher than the previous further analysis of the 2011 demographic and health survey in Ethiopia, 15.3%. Possible explanations could be due to the increasing trend in the use of substances such as alcohol [20] and cigarettes [36] in Ethiopia, and prevalent internet addiction [37] in the region might also potentiate the use of substances such as khat. In addition, this study demonstrated that 35.6% of men in Ethiopia consumed alcohol. This finding is in line with a study conducted in Ethiopia [20, 38], but was at odds with findings in Morocco [39], Bangladesh [40], and the USA [41]. This variation could be due to the difference in instruments used to measure alcohol use, media advertising, and socioeconomic differences.

Khat chewing alone, alcohol use alone, and both khat and alcohol use were associated with multiple factors at the individual and community levels. At individual level: regarding age group, younger men, 15–29 years and 30–49 years increased odds of khat, alcohol and dual use compared to older men, 45–59 years. Previous reports from East Africa [42], Saudi Arabia [43], and India [44]) also confirmed that younger people are more likely to indulge in khat and alcohol, alone or in combination. The higher prevalence of khat and alcohol consumption in younger peoples emphasizes the importance of implementing prevention strategies for this age groups.

This study revealed that cigarette smokers were more likely to use khat. Other researchers have reported a positive associations between these two substances [45]. In one only men study half of daily smokers said they smoke cigarette to increase the effects of khat [46]. The odds of khat and alcohol use alone or in combination were higher in working men than men who were not working. Studies in East Africa [42] and Korea [47] have reported similar findings. The possible reason could be working men are more prone to work-related stress, which might lead to unhealthy coping behavior such as substance use, including khat.

Despite multiple studies have shown that uneducated people are more likely to be affected by khat and alcohol [48–50], our study reported the reverse. Thus, men with higher educational level were more likely to use alcohol, and alcohol and khat in combination. This result is in line with other studies [42, 51, 52]. This could be explained by educated peoples being largely younger, who are more vulnerable to the use of these substances due to peer pressure, curiosity, and a means to relieve stress [53].

With regard to marital status, divorced males were less likely to use khat and alcohol, which points to the probable readiness of people to form new lifestyles and increased resistance to different life challenges, including substance use. In addition, married males had lower odds of alcohol and khat dual use. Other studies also confirmed this finding [42, 54]. Due to limited spare time, sense of commitment, and partners’ influence on marriage, married males are less likely to use alcohol and khat.

This study demonstrated that internet use and TV watching were protective factors for khat use. Another study showed that peoples who used the internet and TV were less likely to use khat. The reason could be individuals who access TV and Internet have increased awareness about side effects of khat use, like depression, anxiety, sleep disturbance, dental problems, and conflict in the family, making them less likely to use khat [19, 55, 56]. Males living in Dire Dawa and Harari regions were more likely to use khat. In fact, these regions are where the country’s largest khat is produced for exports and national consumption [57], which profoundly affects the khat chewing practices of the surrounding population. Previous studies confirmed the specific areal burden of khat chewing [58].

Additionally, we found that males in the Amhara and Tigrai region had 3.49 and 2.7 higher odds of drinking alcohol as compared to males living in Addis Ababa. This finding is consistent with earlier findings [13, 59]. Furthermore, living in urban areas decreased the risk of alcohol use by 45% compared with urban areas, which is in line with previous findings [60]. Similarly, males living in certain regions, such as Afar, Benishangul, Oromia, SNNPR, Somalia, and Tigray are less likely to use both khat and alcohol as compared to Addis-Ababa. Differences in alcohol usage among regions could be due to the lower developed nature of these regions as compared to Addis Ababa, which makes them less accessible to alcohol products, cultural differences amongst regions, and socio-economic variations. This implies that policies and programs on alcohol use prevention need to pay special attention to males living in urbanized areas, such as Addis Ababa.

Rich communities (wealth index at the community level) compared to poor communities decreased the odds of alcohol drinking by 33%. This finding is in agreement with another study [61]. This may be partly explained by poor communities tend to use more alcohol than rich communities as a coping mechanism to reduce stress arising from financial challenges. Protestants, Muslims, and others religion follower decreased the likelihood of alcohol drinking by 99%, 96%, and 65% as compared to Orthodox religion followers. This finding is consistent with previous findings (51.6%) [30, 32]. This might be because drinking alcohol is culturally accepted in Ethiopian orthodox Christian followers.

## Conclusions

The prevalence of dual alcohol and khat use is of concern. Dual use is much lower than either Alcohol or Khat use. Alcohol is by far the most common substance used among male adults. In the final multinomial multilevel model, age of the participant, age of head of household, educational level, marital status, internet use, smoking, number of family members, religion, occupation, and use of TV were associated with dual alcohol and khat use at the individual level. Given this finding, it is wise to implement interventions on modifiable factors, such as educational level, smoking, and marital status. In addition, poor communities and urban areas need special attention to control the prevalence of alcohol and khat consumption.

## Supporting information

S1 File The minimal anonymized dataset. (XLSX)

## Abbreviations

AOR: adjusted odds ratio
CI: confidence interval
EDHS: Ethiopian demographic and health survey
EA: Enumeration areas
GSEM: Generalized structural equation modelling
SNNPR: Southern nations, nationalities, and people of Ethiopia
SUDs: Substance use disorders
WHO: World health organization
